# Hydatidosis of the Pelvic Cavity: A Big Masquerade

**DOI:** 10.1155/2008/782621

**Published:** 2008-08-28

**Authors:** Peyman Varedi, Seyed Reza Saadat Mostafavi, Rambod Salouti, Daryoush Saedi, Seyed Ali Nabavizadeh, Kaveh Samimi, Tahereh Larijani, Mohsen Darabi, Seyed Mehdi Mousavi, Ahmad Ostadali Makhmalbaf

**Affiliations:** ^1^Department of Radiology, School of Medicine, Iran University of Medical Sciences, Tehran 1445613131, Iran; ^2^Department of Radiology, School of Medicine, Shiraz University of Medical Sciences, Shiraz, Iran; ^3^Department of Pathology, School of Medicine, Iran University of Medical Sciences, Tehran 1445613131, Iran

## Abstract

We report and discuss a case of primary hydatidosis of the pelvic cavity in a woman who presented with severe weight loss and abdominal pain. This unusual presentation was initially considered as a tumor process until surgical exploration and microscopic studies confirmed the diagnosis. The gynecologists should be aware of possibility of primary hydatid cyst of the pelvic cavity and should be considered in the differential diagnosis of cystic pelvic masses, especially in areas where the disease is endemic.

## 1. INTRODUCTION

Hydatid disease is a parasitic infection caused by Echinococcus, which is endemic in farming areas. It primarily involves the liver and presents as the gastrointestinal manifestations such as abdominal pain, hepatomegaly, anorexia, vomiting, and jaundice. The primary involvement of the pelvic cavity is a very rare entity and patients usually present with pressure symptoms affecting the adjacent organs [1–5]. Herein, we report this interesting case that presented differently and also discuss the significance of the awareness of physicians about this rare but well-documented disease.

## 2. CASE REPORT

**Figure 1:**
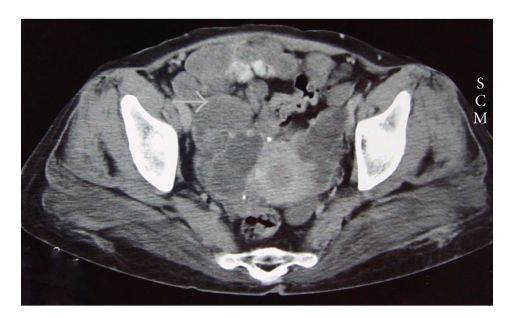
Axial pelvic CT scan demonstrates multiple cystic masses (arrow) in the pelvic cavity and also the uterus (arrowhead).

**Figure 2:**
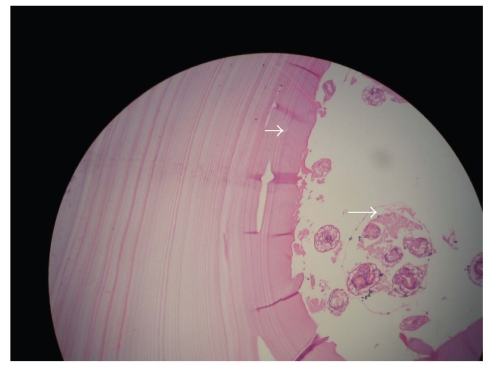
Microphotograph of the lesion reveals laminated membrane (large arrow) and the scolices (small arrow).

A 34-year-old woman was admitted to our center with complaints of lower abdominal pain, anorexia, constipation, and significant weight loss (more than 10 Kg during 4 months). There was no history of fever, nausea, or vomiting. Habitual and occupational history was uneventful. She had 3 normal full-term deliveries. As the patient had severe constipation and other gastrointestinal symptoms, colonoscopy had been carried out in another center with normal findings. On pelvic examination a nontender, uniformly cystic mass approximately 28-week size was palpated. Speculum examination was normal. Laboratory investigations including blood sugar, blood urea nitrogen, creatinine, liver enzymes, and urinalysis were normal and stool occult blood was negative. Pregnancy test was negative. Complete blood count was normal except mild anemia (Hb: 7 gr/dL). Chest radiograph was also normal. On transabdominal ultrasound, multiple anechoic masses were demonstrated in the pelvic and uterine cavities. For further delineation of the cystic masses and disease extension in the pelvic cavity, computed tomography (CT) scan was performed, that revealing multiple cystic space-occupying lesions in the pelvic cavity, involving the uterus, broad ligament, and adnexa (Figure 1). The patient underwent an exploratory laparotomy under general anesthesia for further diagnosis of the cystic mass, and ruling out of the genitourinary malignancy. Preoperative laboratory evaluation including HIV antibody, VDRL test, HBS antigen, and HCV antibody assay were normal. Additionally, whole body bone scan, chest and abdominal CT scan were also performed that revealed unremarkable results. On the 10th day of her admission after obtaining the informed consent, the patient underwent surgical exploration. At the operation, numerous ovarian and paraovarian cystic masses densely adhered to the uterus, to the pelvic side wall, and to the fallopian tubes were demonstrated. One of the cysts was removed completely and sent to the laboratory and this surgical specimen was diagnosed as the hydatid cyst. Due to extensive involvement of the uterus, adnexa, and broad ligament, complete removal of the cysts and preservation of the all reproductive organs was impossible; furthermore complete removal of the cysts due to the high risk of rupture could be life threatening, so after obtaining the signed consent we decided to perform removal of the cysts of the right adnexa and uterus with preservation of them and left side salpingoophorectomy. The rest of the abdomen and pelvis were free of pathology. The abdomen was then carefully irrigated with isotonic saline. Microscopic examination disclosed the scolices of *Echinococcus granulosis* with adjacent laminated membrane and confirmed the diagnosis (Figure 2). The patient recovered uneventfully and was discharged on the 11th postoperative day. Albendazole (800 mg per day) as adjuvant therapy was administered for 4 months postoperatively. Blood count and liver transaminases were checked during the course of therapy which showed normal results. At 6-month follow-up the patient was doing well with no detectable abnormality on follow-up ultrasound.

## 3. DISCUSSION

Hydatid disease is a parasitic disease caused by the larval stage of the tapeworm *Echinococcus granulosus*. While liver and lung are the most commonly affected areas in adults, hydatid cysts may develop in almost any part of body [1, 2, 4]. Primary hydatidosis of the pelvic cavity is very rare but well documented in endemic areas such as the Mediterranean countries, South America, the Middle East, and Australia [1, 3]. Our patient is the first reported case of primary hydatidosis of the pelvic cavity from Iran and underlines the difficulties in the diagnosis of cases with striking resemblance to malignant disease of the reproductive tract. To our knowledge, the primary involvement of the pelvic cavity usually presents with pressure symptoms affecting the adjacent organs [5] and this manifestation has been reported exceedingly rare in the English literature so far [3]. Diagnosis of the hydatid cyst is mainly on the basis of serologic tests and/or ultrasonography and CT scan. However, surgical exploration may be necessary for definitive diagnosis [5–8]. Previous history of hydatid cysts or exposure to dog and farm animals should raise the suspicion of this diagnosis. Following the surgical diagnosis in our patient, we inquired about such a history in our patient but revealed no identifiable exposure. Specific serological methods using specific antigens, especially native AgB, have been recommended for proper diagnosis because the serological tests using crude antigens are sensitive, but their specificity is not satisfactory. In one study, the enzyme-linked immunosorbent assay (ELISA) system is much more specific in detecting antihydatid cyst antibody than countercurrent immunoelectrophresis (CCIEP), while CCIEP is more sensitive in detecting antihydatid cyst antibody [9]. Interestingly, we carried out the CCIEP assay immediately after the confirmatory result of the pathology that was positive for hydatid cyst therefore serologic tests in the patients with suspicious diagnosis may render the diagnosis of the hydatid cyst. Ultrasound is an important imaging modality for hydatid disease and may clearly demonstrate the floating membranes, and daughter cysts characteristically seen in purely cystic lesions. The ultrasonographic findings range from purely cystic lesions to a completely solid appearance [10]. In another study, CCIEP could detect only 62.0% of cases, whereas the pathology and ultrasound results were positive for 96.3% of cases. This study emphasized the usefulness of ultrasound and suggested that CCIEP may be useful for diagnosing cystic *Echinococcus* only in doubtful cases [11]. CT and magnetic resonance imaging (MRI) play a key role in recognizing the complications such as rupture and infection of cysts associated with hydatid disease. We believe that CT scan—because of its capability for better evaluation of the cystic masses, and better demonstration of their extension in the pelvic cavity as well as excellent depiction of the visceral organs involvement—is superior to the ultrasonographic examination. The scan in suspected hydatid disease should include the whole abdomen from liver to pelvis and a chest radiograph should also be obtained. Skin tests, complement fixation, blood eosinophil count, and indirect hemagglutination tests can also be used for diagnostic purpose however their tendency toward the false-positve results limits their validity. The gold standard test for diagnosis of hydatidosis is microscopic examination that shows the laminated membrane and scolices [1–4, 6–9]. Surgical removal is the optimal treatment. Complete removal of the parasitic cysts and fluid is the major advantage of the surgery. Single cysts are easy to excise but due to the risk of adhesions, excision of the multiple cysts may be difficult and even impossible [1–6]. We agree with this comment which emphasizes that in the younger women, even in those with multiple cysts, every effort should be made to preserve reproductive organs [2]. As previously stated in this case, extensive involvement and hard adhesion of the adnexa to the adjacent cysts of the broad ligaments forced us to perform left-sided salpingoophorectomy. Another important problem is dealing with intraoperative spillage of the cyst contents that contain protoscolices and can disseminate in tissue and grow new cysts. Furthermore, acute anaphylactic reaction may ensue secondary to the spilled cyst fluid therefore spillage should be avoided by all means [2, 4–8]. The treatment of the recurrence and complications is very difficult and definition of the best option needs multidisciplinary approach therefore medical therapy should be applied postoperatively in the patients with multiple cysts and multiple initial locations. The clinical manifestation of our case such as severe weight loss, as well as the large multicystic mass of the pelvic cavity erroneously points us to the diagnosis of the ovarian malignancies. The current case underlines the possibility of the striking resemblance between the clinical and radiological manifestation of the hydatid cyst and malignant disease of the reproductive organs which may make the correct preoperative diagnosis very difficult. The tumor markers were not checked in our case however lack of the other evidences of the metastasis to the visceral organs in the chest and abdominal CT scan and normal result of the bone scan was the useful guidelines against the diagnosis of the ovarian malignancy. Although normal results of the tumor markers such as CEA and CA-125 may be found in the patients with genitourinary malignancies especially ovarian cancer, we note that these markers should be evaluated in the patients with the suspicious diagnosis of the genitourinary malignancies. In conclusion, primary hydatidosis of the pelvic cavity should always be considered in the differential diagnosis of any tumor-like growing mass even in the absence of accompanying involvement of liver or other visceral organs. We believe that in the cases like our patient, the most important factor in diagnosis of hydatid disease of the pelvic cavity is the high index of suspicion about its possibility which can provide the accurate diagnosis and prevent the erroneous treatment. The other important consideration is the accidental rupture of hydatid cyst during surgery which may be life threatening, therefore preoperative diagnosis of this rare lesion is very important. The patient's history as well as the serologic tests may yield important clues about the diagnosis, furthermore the radiologist's familiarity with the imaging findings of the disease is very important for earlier diagnosis and an appropriate treatment.
